# A Case of Excision of the Tongue for Epithelioma

**Published:** 1890-08

**Authors:** C. D. Roy

**Affiliations:** Senior Assistant, Charity Hospital, N. Y.


### A CASE OF EXCISION OF THE TONGUE FOR EPITHELIOMA.

Reported by C. D. Roy, M. D., Senior Assistant, Charity Hospital, N. Y.

Upon coming into the hospital the patient gave the following history: Ten years ago he had the initial lesion of syphilis upon his penis, soon followed by all the secondary manifestations of that disease. He was treated for that affection with apparent recovery, having had no return of the symptoms in the past five years. Had always enjoyed good health, but was addicted to some of the vices of the present age. He was an inveterate smoker, and preferred the pipe to any other form of smoking. In fact he lived continuously with the stem of it in his mouth. Was addicted to the excessive use of strong drinks for a number of years. A short time before his admission he was struck on the side of the tongue while in a drunken stupor. This produced an abrasion which gradually grew larger, and about a month after the injury he noticed a lump under his lower jaw. The pain of his tongue was incessant, and on his admission the diagnosis of epithelioma of the tongue was made. Dr. J. E. Kelly, the visiting surgeon, decided that an extirpation of the tongue was the only hope for the patient. The latter was of heavy frame, forty-four years old, and single. The patient having been placed under an anæsthetic the operation was commenced by tying the lingual arteries to prevent as much hemorrhage as possible. An incision was then made in the median line through the skin, fascia and muscular tissue down to the bone substance of the inferior maxilla. Having done this a narrow saw was used to separate this bone at the symphysis, thus to pull aside the two lateral halves and making the tongue much more accessible. A strong silk thread was passed through the tip of the tongue in order to keep that organ pulled forward. A ligature was then passed around the base of the tongue and that organ removed by cuts from a pair of curved scissors. Not much hemorrhage was experienced and that which did exist was checked by the application of hot water. The two halves of the inferior maxilla were then united by a wire suture and the superficial wound closed with silk. Several of the glands in the neck were found enlarged, and these Dr. Kelly removed in toto. The mouth was washed out several times during the day with a mild solution of permanganate of potash, which was the only dressing used to that organ. As alimentation was necessary for the life of the patient an opening was made into the æsophagus and a tube introduced therein by means of which liquid food was introduced into the alimentary canal. This latter process was continued for little over a week, when liquid and soft food could be placed upon the stump and thus introduced into the alimentary tract. The sense of taste was somewhat impaired and the pronunciation of the dental and labial letters imperfect, but otherwise the patient made excellent recovery. An amusing incident occurred during the morning after the operation which is worthy of mention, especially in a Southern journal. The patient had fought on the Confederate side during the late war and he was aware that the House Surgeon then in charge was the only southerner at the operation. On the morning alluded to he asked for a pencil and a piece of paper, on which he wrote the following to the House Surgeon: "I guess you and I were the only 'Johnny Rebs' at the fun on yesterday." He has since been engaged by a museum, as the only living man in existence who can talk with his tongue cut out.

